# MRI proton density fat fraction for estimation of tumor grade in steatotic hepatocellular carcinoma

**DOI:** 10.1007/s00330-023-09864-x

**Published:** 2023-06-27

**Authors:** Patrick Arthur Kupczyk, Darius Kurt, Christoph Endler, Julian Alexander Luetkens, Guido Matthias Kukuk, Florian Fronhoffs, Hans-Peter Fischer, Ulrike Irmgard Attenberger, Claus Christian Pieper

**Affiliations:** 1 Department of Diagnostic and Interventional Radiology, University Hospital Bonn, Venusberg-Campus 1, 53127 Bonn, Germany; 2 Quantitative Imaging Lab Bonn (QILaB), Bonn, Germany; 3 Department of Radiology, Kantonsspital Graubünden, Loestrasse 170, 7000 Chur, Switzerland; 4 Institute of Pathology, University Hospital Bonn, Venusberg-Campus 1, 53127 Bonn, Germany

**Keywords:** Hepatocellular carcinoma, Liver, Magnetic resonance imaging, Neoplasm grading

## Abstract

**Objectives:**

Image-based detection of intralesional fat in focal liver lesions has been established in diagnostic guidelines as a feature indicative of hepatocellular carcinoma (HCC) and associated with a favorable prognosis. Given recent advances in MRI-based fat quantification techniques, we investigated a possible relationship between intralesional fat content and histologic tumor grade in steatotic HCCs.

**Methods:**

Patients with histopathologically confirmed HCC and prior MRI with proton density fat fraction (PDFF) mapping were retrospectively identified. Intralesional fat of HCCs was assessed using an ROI-based analysis and the median fat fraction of steatotic HCCs was compared between tumor grades G1-3 with non-parametric testing. ROC analysis was performed in case of statistically significant differences (*p* < 0.05). Subgroup analyses were conducted for patients with/without liver steatosis and with/without liver cirrhosis.

**Results:**

A total of 57 patients with steatotic HCCs (62 lesions) were eligible for analysis. The median fat fraction was significantly higher for G1 lesions (median [interquartile range], 7.9% [6.0─10.7%]) than for G2 (4.4% [3.2─6.6%]; *p* = .001) and G3 lesions (4.7% [2.8─7.8%]; *p* = .036). PDFF was a good discriminator between G1 and G2/3 lesions (AUC .81; cut-off 5.8%, sensitivity 83%, specificity 68%) with comparable results in patients with liver cirrhosis. In patients with liver steatosis, intralesional fat content was higher than in the overall sample, with PDFF performing better in distinguishing between G1 and G2/3 lesions (AUC .92; cut-off 8.8%, sensitivity 83%, specificity 91%).

**Conclusions:**

Quantification of intralesional fat using MRI PDFF mapping allows distinction between well- and less-differentiated steatotic HCCs.

**Clinical relevance:**

PDFF mapping may help optimize precision medicine as a tool for tumor grade assessment in steatotic HCCs. Further investigation of intratumoral fat content as a potential prognostic indicator of treatment response is encouraged.

**Key Points:**

• *MRI proton density fat fraction mapping enables distinction between well- (G1) and less- (G2 and G3) differentiated steatotic hepatocellular carcinomas.*

• *In a retrospective single-center study with 62 histologically proven steatotic hepatocellular carcinomas, G1 tumors showed a higher intralesional fat content than G2 and G3 tumors (7.9% vs. 4.4% and 4.7%; p* = *.004).*

• *In liver steatosis, MRI proton density fat fraction mapping was an even better discriminator between G1 and G2/G3 steatotic hepatocellular carcinomas.*

## Introduction

Over the last decades, imaging of hepatocellular carcinoma (HCC) has evolved from a mere tool for gaining adjunct information on tumor localization and the extent to the non-invasive gold standard for HCC diagnosis, challenging the status of liver biopsy. According to current guidelines, HCC in cirrhotic livers can be diagnosed with certainty by imaging if the suspicious lesion meets clearly defined criteria [1]. Particularly magnetic resonance imaging (MRI) of the liver has proven to be the imaging modality of choice for the detection and characterization of HCC. Besides typical diagnostic hallmarks, such as arterial phase enhancement and wash-out in portal venous or late venous phase, less specific characteristics have occasionally been implemented in guidelines. In LI-RADS [2], several ancillary features favoring the diagnosis of HCC have been defined; one of them is the presence of intralesional fat. However, this emphasis on fat deposition in HCCs is backed up by only a few histopathological and radiological studies implying a relationship between the presence of intratumoral fat and the degree of differentiation [3, 4], some of them even proposing a prognostic value of intralesional fat detection using MRI [5, 6]. As histopathological tumor grade has been shown to be a predictor of therapeutic outcome and recurrence in HCC [7–9], a non-invasive estimation of tumor grade may serve as a prognostic tool and thus guide therapeutic decisions, especially when tissue sampling is not necessary or possible. Although tumor grade is currently not part of the widely accepted BCLC staging system, this feature could potentially contribute to the evolution of precision medicine by reflecting biological and molecular characteristics that influence treatment response [10].

The most widely used MR technique for fat detection (i.e., chemical shift imaging) exclusively allows a qualitative assessment of intralesional fat [11], whereas MRI proton density fat fraction (PDFF) mapping enables robust and reliable quantification of hepatic fat content (e.g., in diffuse liver disease) and can be easily embedded into standard liver imaging protocols [12–14]. However, as its significance for the characterization of focal liver lesions is still unknown, the question arises whether PDFF has additional diagnostic value beyond the detection of intratumoral fat in HCC, and may possibly serve as a surrogate for tumor grade.

The aim of this study was therefore to evaluate a potential association between intralesional fat content, as determined by PDFF, and histopathologic tumor grade in steatotic HCCs.

## Material and methods

This retrospective study was approved by the local institutional review board and informed consent was waived for all subjects.

### Study population

To identify eligible patients, examination data from clinically indicated abdominal MRI scans that routinely included an mDixon-based fat quantification sequence for the liver performed between December 2013 and July 2020 were first extracted from the institutional Picture Archiving and Communication System (IMPAX EE, AGFA HealthCare). These data were then matched with the electronic database of the local Institute of Pathology and screened for lesions with histological confirmation of HCC within 30 days of imaging, either by biopsy or by surgical resection. HCC lesions with previous treatment as documented in the hospital´s medical information system were excluded. Eventually, ROI-based analysis of the fat quantification maps (see below) was used to filter out steatotic HCCs by excluding lesions without detectable fat. Clinical and laboratory data were retrieved from the institutional clinical information system to further describe the patient population under investigation.

### Tumor pathology

Data regarding HCC diagnosis, lesion location, method of tissue sampling, and histopathological tumor grade according to the WHO criteria (well [G1]-, moderately [G2]-, and poorly [G3] differentiated) were obtained for each steatotic HCC from the electronic database of the Institute of Pathology. The available tissue sections of the lesions obtained by resection were reviewed by a pathologist with more than 10 years of experience in the field of liver pathology (F.F.). He semiquantitatively estimated the amount of intralesional fat in steatotic HCCs using a 4-point scale (grade 0, very mild to none: <  = 2%; grade 1, mild: > 2─5%; grade 2, moderate: > 5─10%; grade 3, severe: > 10%).

### MR-imaging and image analysis

MRI examinations were performed on either a 1.5-Tesla (Ingenia, Philips Healthcare) or a 3 Tesla (Ingenia, Philips Healthcare) MR system. The MRI protocols consisted of standard sequences for abdominal imaging, including T2-weighted sequences with a radial readout, contrast-enhanced dynamic liver imaging, chemical shift imaging, and a six-echo 3D gradient-echo sequence (mDixon) in axial orientation for proton density fat fraction (PDFF) and T2* mapping. The latter has been extensively studied in diffuse liver disease and the technique is described in detail elsewhere [15, 16]. For contrast-enhanced dynamic liver imaging, gadobutrol (Gadovist, Bayer HealthCare) was administered at a dose of 0.1 mmol per kg body weight.

**Fig. 1:**
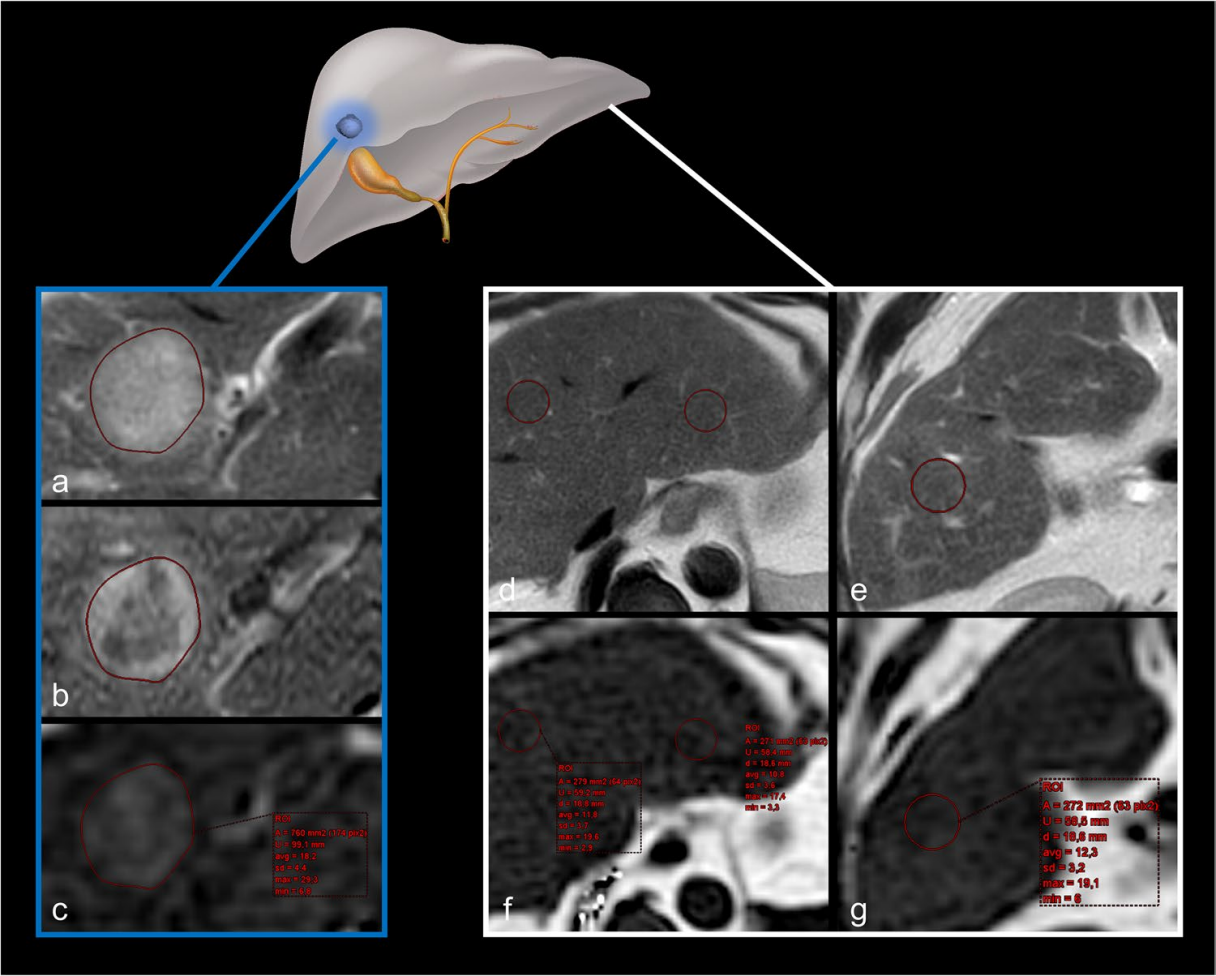
Illustration of ROI placement in an HCC (blue frame) and in liver parenchyma (white frame) to measure proton density fat fraction. The ROIs were first placed in slices of conventional T2-weighted (**a**, **d**, **e**) or ─ if degraded by severe artifacts ─ of contrast-enhanced T1-weighted images (**b**) before being copied to the corresponding locations on the PDFF maps (**c**, **f**, **g**). ROI = region of interest, HCC = hepatocellular carcinoma, PDFF = proton density fat fraction

Image analysis was performed by two board-certified radiologists with 10 years (P.A.K.) and 12 years (C.C.P.) of experience in abdominal MRI, respectively. Both readers were blinded to histologic tumor grade and clinical data, but not to tumor location as documented in the biopsy or surgery reports. In cases of multifocal HCC or histological sampling of more than one lesion, the pathologic report was matched with the complete image dataset or the biopsy images to unequivocally identify the sampled lesion. In addition to measuring the maximum tumor diameter, fat concentration in HCC lesions was quantified independently by both readers on the generated PDFF maps using regions of interest (ROIs). Due to its robustness to motion artifacts and a reliable delineation of lesions, ROIs were initially placed in the axially oriented T2-weighted sequence in the slices, where the greatest lesion diameter had been determined, covering the entire circumference of the lesion. Attention was paid to avoiding large vascular structures and possible tumor capsules. In cases of uncertain lesion margins, the contours were cross-checked using dynamic post-contrast images. The ROIs were then copied to the PDFF maps and the water-only outputs of the mDixon sequence, and the correspondence of locations was verified visually. In cases of obvious misregistration, the ROIs were manually adjusted accordingly. Measurements of fat fraction were extracted as the average of the percentage derived from each ROI, as exemplified in Fig. 1. Each reader performed the measurement three times per lesion and averaged the results. To assess liver steatosis, two ROIs were placed in the right liver lobe and one ROI in the left liver lobe in the same image plane, avoiding large vessels and focal lesions. The average parenchymal fat content was calculated based on these ROIs (see also Fig. 1). Although these PDFF measurements were taken by both readers to assess inter-reader reliability, only the data from the more experienced reader (C.C.P.) were considered for further statistical analyses and presented in detail.

To assess a possible influence of the iron content in HCCs or in the liver parenchyma on the PDFF measurements, the experienced reader also performed ROI-based measurements on the T2* maps in both lesions and parenchyma in an analogous manner. The outputs were converted to R2* (1/T2*), and liver iron content and lesion iron content were calculated using recently published calibration curves for the estimation of liver iron content by R2* relaxometry [17].

The image datasets of the HCCs classified as steatotic were also reviewed by the more experienced of the two readers for the presence of typical imaging features of HCCs (i.e., arterial phase enhancement and wash-out).

### Definitions

Following Hong et al [18], who consider MR-derived PDFFs below 2.2% as image noise, lesions with readings above this threshold, as determined by the radiologist with 12 years of experience in abdominal MRI (C.C.P.), were classified as steatotic.

Liver steatosis was defined as an average fat fraction in liver parenchyma > 5.0% (6,11,12).

### Statistical analysis

Statistical analyses were performed using IBM SPSS Statistics, version 26.0 (IBM Corp.), Stata Statistics software, version 14.2 (Stata Corp.), and Prism 9.0 (GraphPad Software Inc.). Normal distribution was checked by using the Shapiro–Wilk test. Descriptive statistics were reported as median with interquartile range (IQR) or mean ± standard deviation for continuous variables, as appropriate, or as counts and percentages for dichotomous variables. The median fat fraction and diameter of the steatotic lesions, as determined by C.C.P., were assessed for all tumor grades and comparisons were performed using the Kruskal–Wallis test and Dunn´s multiple comparisons test. *p* values < 0.05 were considered significant. In case of significant differences between tumor grades, the diagnostic performance of PDFF as a discriminator between groups was evaluated using ROC analysis, and the optimal cut-off value was determined using Youden’s index. In an analogous fashion, subgroup analyses were performed for subjects with/without liver steatosis and with/without liver cirrhosis. The proportions of steatotic HCCs that had the typical imaging hallmarks of HCC were compared between tumor grades with the chi-square test. Inter-reader agreement regarding PDFF measurements was analyzed by calculation of the intraclass correlation coefficient. The correlation of MR-derived PDFF and histological semiquantitative assessment of fat content in resected steatotic HCCs was evaluated using the Spearman rank correlation, and the different groups were compared using the Kruskal–Wallis test.

## Results

### Patient and lesion characteristics

**Fig. 2:**
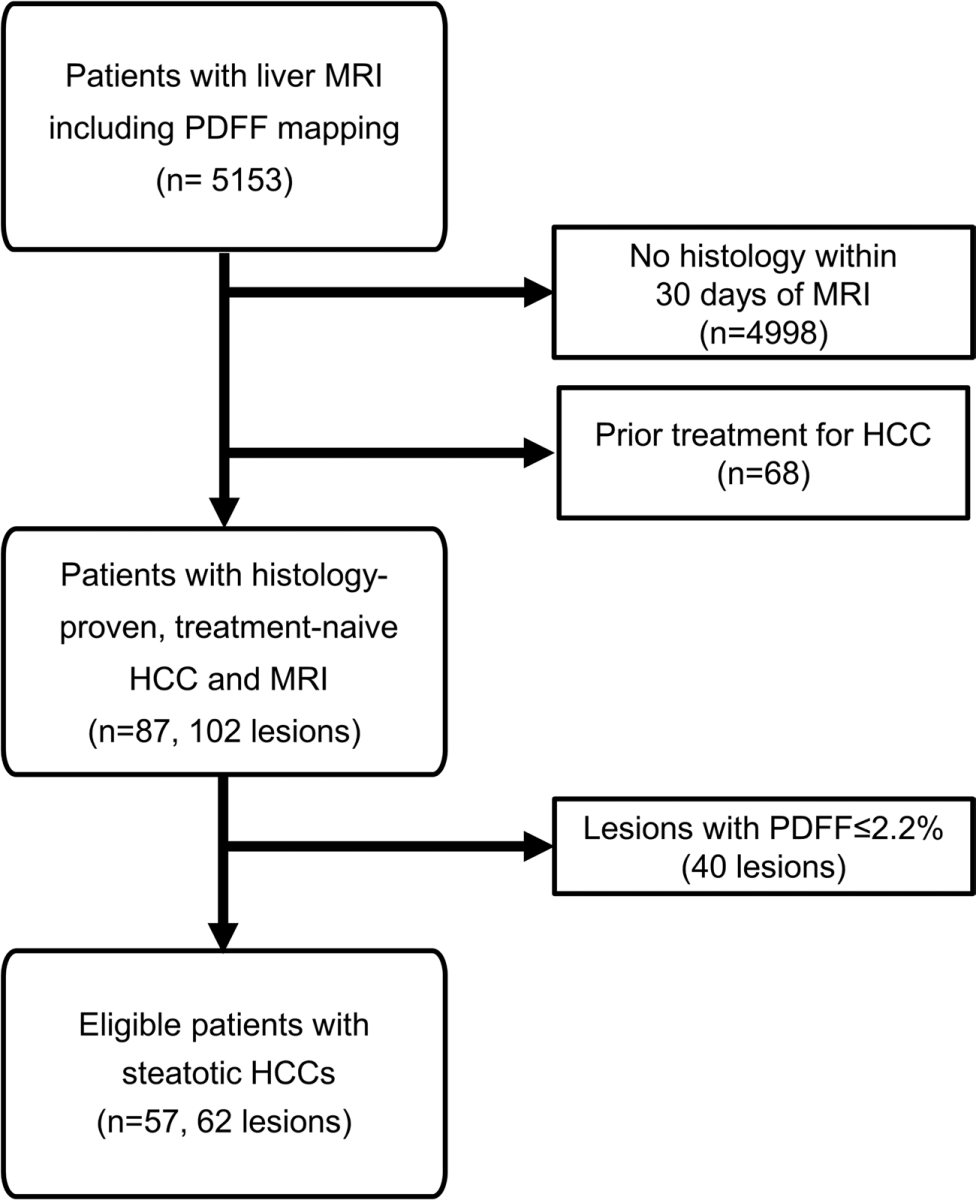
Flowchart of patient selection for the study. PDFF = proton density fat fraction, HCC = hepatocellular carcinoma

Of 5153 patients who underwent liver MRI with PDFF mapping, 155 (3%) were diagnosed with HCC by pathology. Further 68 patients were excluded due to previous treatment for HCC. Of the remaining lesions, 40 HCCs (46%) without detectable intralesional fat were screened out using PDFF mapping. Ultimately, 57 patients (mean age, 68 years ± 9 [standard deviation], 51 men [89%]) for a total of 62 histologically confirmed steatotic HCC lesions were included in the final analyses. A flowchart for patient and lesion selection is given in Fig. 2. Five of 57 patients (9%) had two lesions, which were examined individually by the pathologist. Of 62 samples, 36 (58%) were gained by resection, 26 (42%) by image-guided biopsy. Of the 62 lesions, 41 were examined at 1.5 Tesla (66%), whereas the remainder 21 lesions were examined at 3 Tesla (34%). Liver steatosis was found in 27 out of 57 patients (47%), whereas 42 of 57 patients (74%) had liver cirrhosis. Twelve of 62 lesions were classified as G1 (19%), while 44 of 62 were G2 lesions (71%) and 6 of 62 were G3 lesions (10%). A more detailed overview of patient and lesion characteristics is given in Table 1.

**Table 1:** General characteristics of the study sample

Variable	Value
Clinical parameters	
Gender	
Male	51 (89)
Female	6 (11)
Age at sampling (y)*	68 ± 9
BMI (kg/m^2^)**	28.3 (25.5─29.9)
Liver steatosis	27 (47)
Liver cirrhosis	42 (74)
Child A	37 (88)
Child B	5 (12)
Child C	0 (0)
Etiology	
Hepatitis C	12 (29)
Ethyl-toxic	10 (24)
NAFLD	6 (14)
Other	5 (12)
Cryptogenic	9 (21)
ECOG	
0	43 (75)
1	14 (25)
≥ 2	0 (0)
MELD score**	9 (7─10)
Biochemical parameters	
Total bilirubin (mg/dL)**	0.7 (0.5─1.2)
AST (U/L)**	49 (32─71)
ALT (U/L)**	49 (26─30)
Albumin (g/L)**	40.7 (34.8─45.2)
INR**	1.1 (1.0─1.2)
AFP (ng/mL)**	8.2 (3.4─54.8)
Distribution of lesions	
Total number	62 (100)
Tumor grade	
G1	12 (19)
G2	44 (71)
G3	6 (10)

Note.**—**Except where indicated, data are absolute frequencies, with percentages in parentheses

^*^Data are means ± standard deviation. **Data are medians, with interquartile range in parentheses. *BMI*, body mass index; *NAFLD*, non-alcoholic fatty liver disease; *ECOG*, Eastern Cooperative Oncology Group; *MELD*, model for end-stage liver disease; *AST*, aspartate transaminase; *ALT*, alanine transaminase; *INR*, international normalized ratio; *AFP*, alpha-fetoprotein; *G1*, well-differentiated lesions; *G2*, moderately-differentiated lesions; *G3*, poorly-differentiated lesions

### PDFF results and tumor grade

**Fig. 3:**
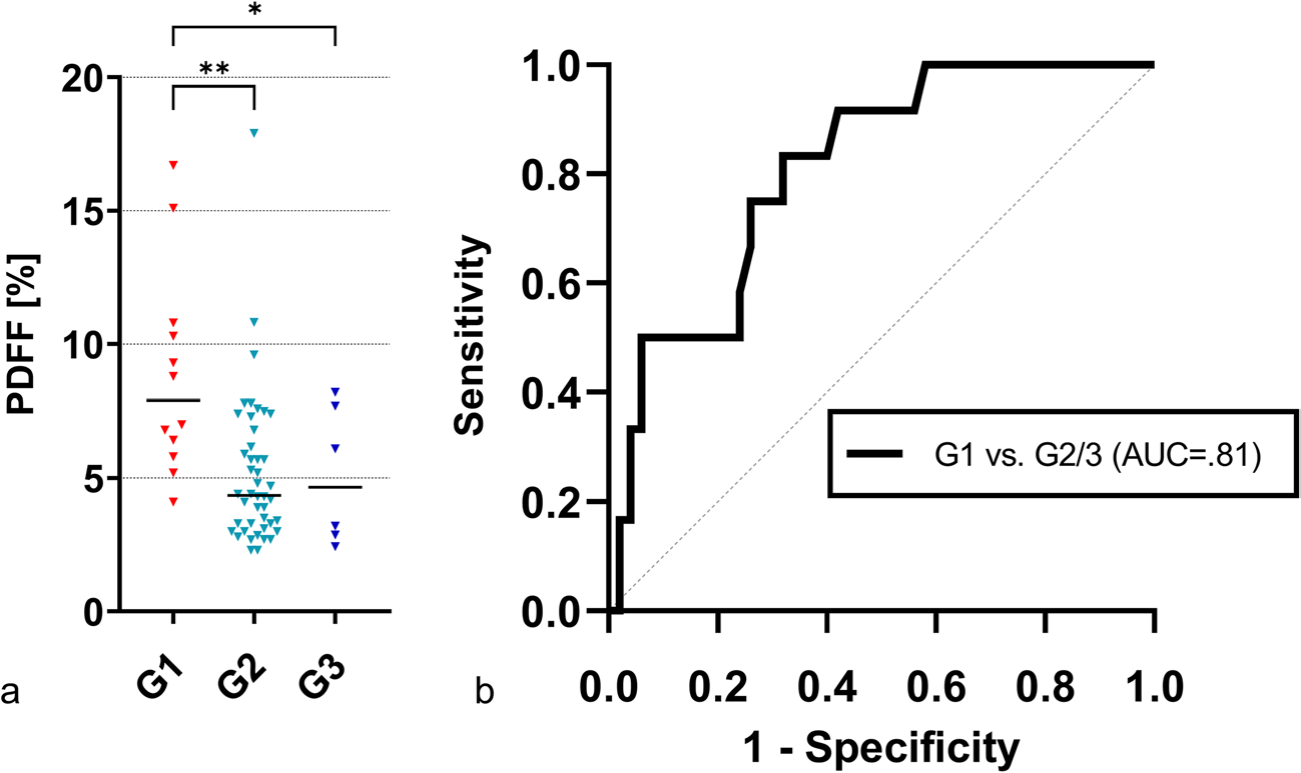
Relationship between intralesional fat content and HCC grade. The measured fat fraction in steatotic HCCs classified as G1 is significantly higher than in G2 and G3 lesions (**a**). ROC analysis highlights intralesional fat fraction as a good discriminator between G1 and G2/3 steatotic HCCs (**b**). * indicates significant pairwise comparison (*p* < .05). ** indicates significant pairwise comparison (*p* < .01). HCC = hepatocellular carcinoma, PDFF = proton density fat fraction, G1 = well-differentiated lesions, G2 = moderately-differentiated lesions, G3 = poorly-differentiated lesions, AUC = area under the receiver operating characteristic curve

**Fig. 4:**
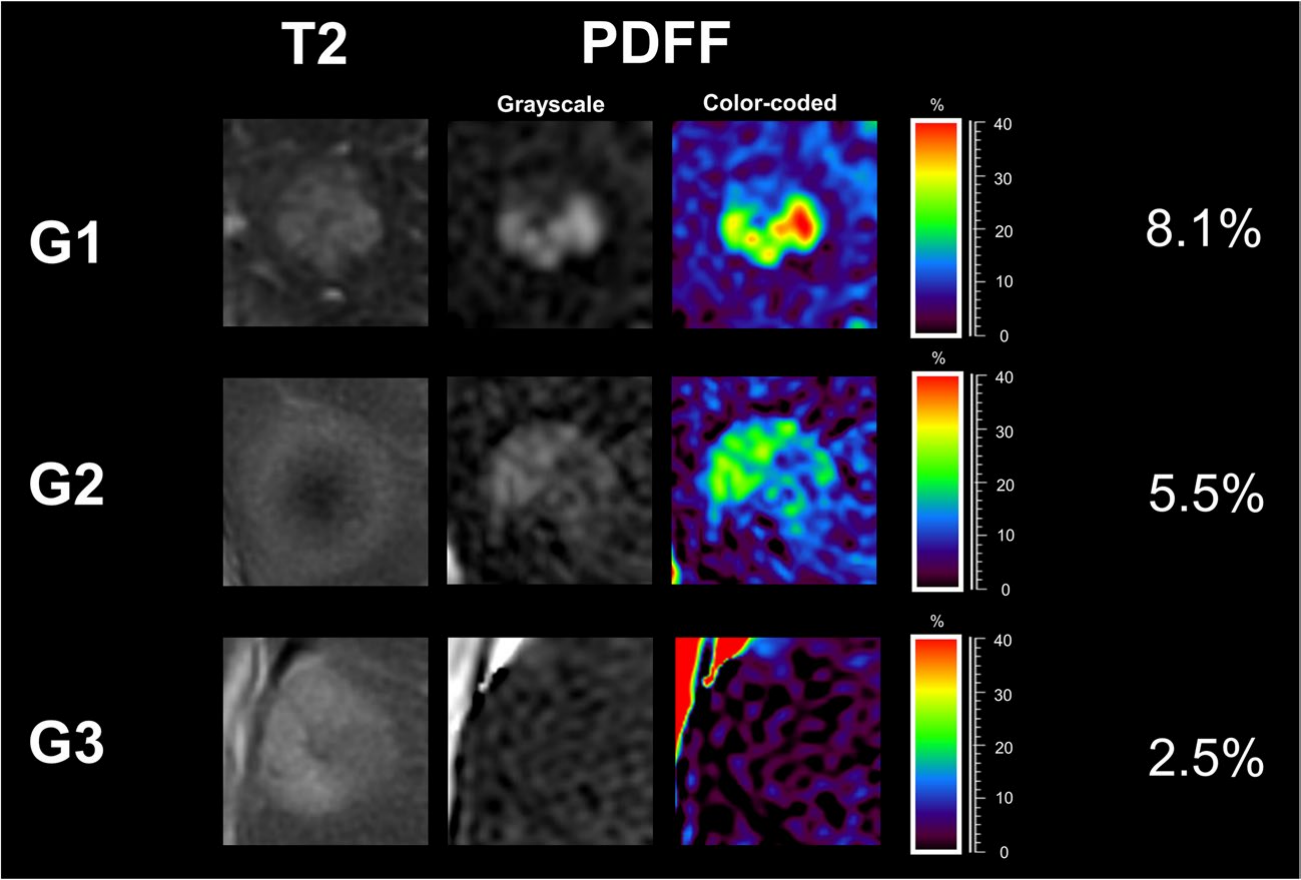
Representative examples of HCC lesions with different histologic tumor grade and their respective MR-derived fat fraction maps (grayscale and color-coded). Note the decrease of average intralesional fat amount with higher tumor grade. HCC = hepatocellular carcinoma, PDFF = proton density fat fraction, G1 = well-differentiated lesions, G2 = moderately-differentiated lesions, G3 = poorly-differentiated lesions

Steatotic HCCs had a median fat content of 5.2% (IQR, 3.3─7.4%). Those classified as G1 were found to have a significantly higher median fat content (7.9%; IQR, 6.0─10.7%) than those categorized as G2 (4.4%; IQR, 3.2─6.6%; *p* = 0.001) and G3 (4.7%; IQR, 2.8─7.8; *p* = 0.036, see also Table 2 and Fig. 3a). No significant differences were found between G2 and G3 lesions in the PDFF measurements (*p* = 0.976). In the ROC analysis, quantified intralesional fat allowed discrimination of G1 and G2/3 steatotic HCCs with an area under the receiver operating characteristic curve (AUC) of 0.81. The determined optimal threshold for the diagnosis of G1 lesions was a PDFF of greater than 5.8% with a sensitivity of 83% (95% confidence interval [CI], 62─100%) and a specificity of 68% (95% CI, 54–81%; Table 3 and Fig. 3b). Representative examples of different HCC grades with their respective PDFF maps are provided in Fig. 4.

**Table 2:** General results on the relationship between MR-derived proton density fat fraction (PDFF), lesion diameter, and histologic tumor grade in steatotic HCCs

Variable	All steatotic HCCs (*n* = 62)	G1 (*n* = 12)	G2 (*n* = 44)	G3 (*n* = 6)	*p* value^1^			
					Kruskal–Wallis test	Dunn’s multiple comparisons test		
						G1 vs G2	G1 vs G3	G2 vs G3
PDFF (%)	5.2 (3.3─7.4)	7.9 (6.0─10.7)	4.4 (3.2─6.6)	4.7 (2.8─7.8)	.004^†^	.001^†^	.036^†^	.976
Diameter (mm)	34.4 (19.0─51.1)	44.3 (36.3─56.9)	31.5 (17.5─44.0)	41.2 (30.0─84.9)	.058	-	-	-

Note.**—**Data are medians; with interquartile range in parentheses. *HCC*, hepatocellular carcinoma; *PDFF*, proton density fat fraction; *G1*, well-differentiated lesions; *G2*, moderately-differentiated lesions; *G3*, poorly-differentiated lesions

^†^Denotes significant values

**Table 3:** Indicators of the diagnostic performance of MR-derived proton density fat fraction (PDFF) to distinguish between well-differentiated (G1) and less-differentiated (G2 and G3) steatotic HCCs in the overall sample, in liver steatosis, and in liver cirrhosis

Variable	AUC	*p* value	Cut-off	Sensitivity (95% CI)	Specificity (95% CI)
All steatotic HCCs (*n* = 62)	.81	.001^†^	5.8%	83% (62─100%)	68% (54─81%)
Steatotic HCCs in liver steatosis (*n* = 28)	.92	.002^†^	8.8%	83% (53─100%)	91% (79─100%)
Steatotic HCCs in liver cirrhosis (*n* = 49)	.79	.003^†^	5.8%	82% (59─100%)	71% (57─85%)

Note.**—***HCC*, hepatocellular carcinoma; *AUC*, area under the receiver operator characteristic curve; *CI*, confidence interval

^†^Denotes significant values

### Steatotic lesions in fatty livers

**Fig. 5:**
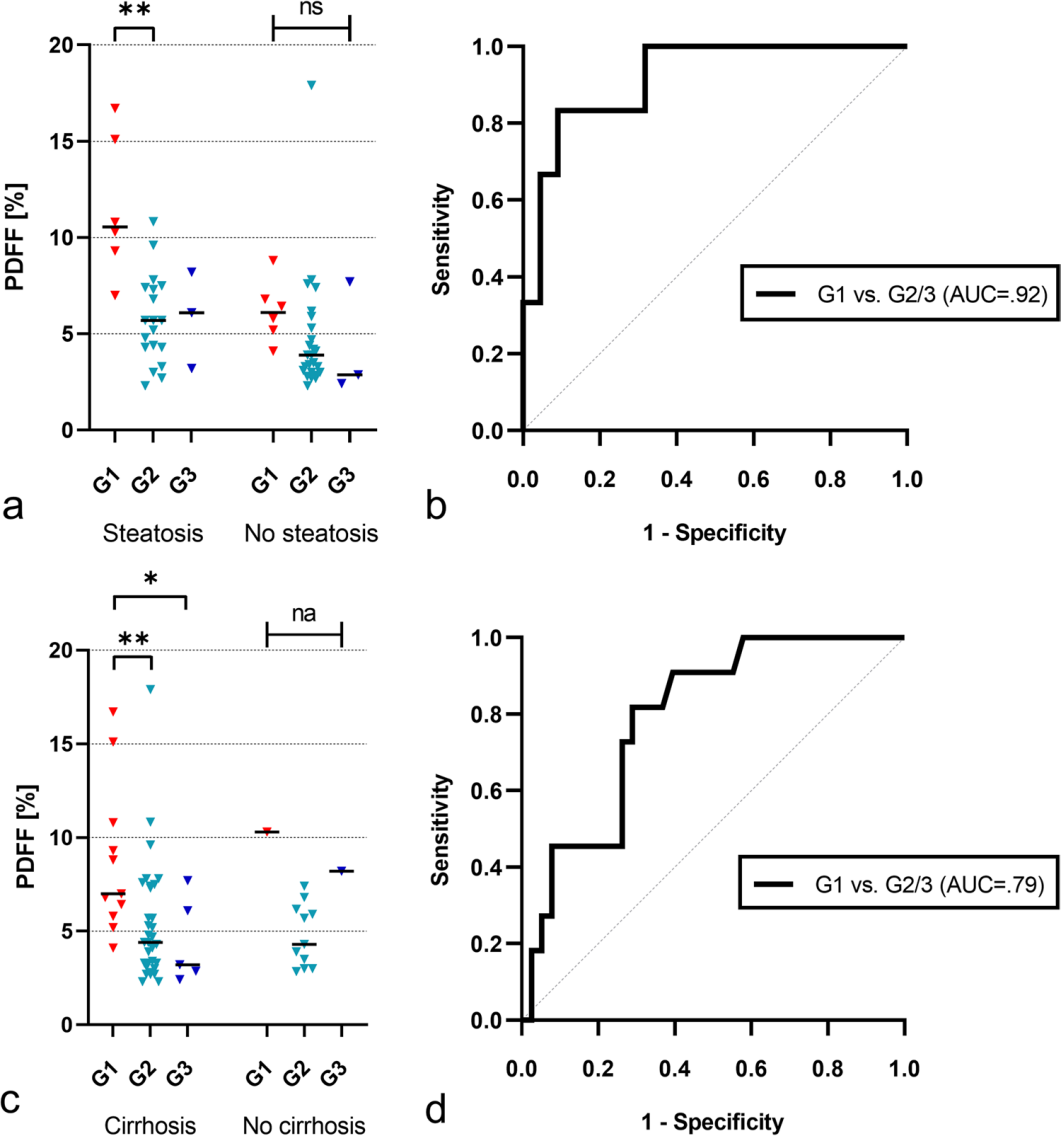
Relationship between intralesional fat content and HCC grade in livers with/without steatosis and with/without cirrhosis. Fat fraction in G1 lesions is significantly higher than in lesser-differentiated HCCs in liver steatosis (**a**) and in liver cirrhosis (**c**). For both steatotic (**b**) and cirrhotic (**d**) livers, ROC analysis shows the excellent diagnostic performance of PDFF in distinguishing between G1 and G2/3 steatotic HCCs. * indicates significant pairwise comparison (*p* < .05). ** indicates significant pairwise comparison (*p* < .01). ns indicates non-significance. na indicates where statistical analysis was not applicable. HCC = hepatocellular carcinoma, G1 = well-differentiated lesions, G2 = moderately-differentiated lesions, G3 = poorly-differentiated lesions, PDFF = proton density fat fraction, AUC = area under the receiver operating characteristic curve

Liver steatosis was observed for 27 of the 57 patients (47%), accounting for a total of 28 HCCs. These were composed of 6 G1 lesions (21%), 19 G2 lesions (68%), and 3 G3 lesions (11%). In this subgroup median fat fraction in steatotic HCCs was even higher (6.5%; IQR, 4.3─9.0%) than in the overall sample, reaching significant differences between G1 (10.6%; IQR, 8.7─15.5%) and G2 tumors (5.7%; IQR, 4.3─7.4%; *p* = 0.002; Table 4 and Fig. 5a). However, we found no evidence of differences between G1 and G3 lesions (6.1%; IQR, 3.2─8.2%; *p* = 0.066). ROC analysis indicated that PDFF distinguishes between G1 and G2/3 steatotic HCCs with an AUC of 0.92 and an optimal cut-off value of 8.8% (sensitivity, 83%; 95% CI, 53─100%; specificity, 68%; 95% CI, 54–81%; Table 3 and Fig. 5b). In the 30 non-steatotic livers, a total of 34 steatotic HCCs were present, including 6 G1 tumors (18%), 25 G2 tumors (73%), and 3 G3 tumors (9%). Despite similar trends of the PDFF values, we found no significant differences in the intralesional fat between groups (*p* = 0.099).

**Table 4:** Subgroup analyses of steatotic HCCs in patients with/without liver steatosis and with/without liver cirrhosis

Variable	All tumor grades	G1	G2	G3	*p* value^1^			
					Kruskal–Wallis test	Dunn´s multiple comparisons test		
						G1 vs G2	G1 vs G3	G2 vs G3
PDFF (%)								
Liver steatosis (*n* = 28)	6.5 (4.3─9.0)	10.6 (8.7─15.5)	5.7 (4.3─7.4)	6.1 (3.2─8.2)	.008^†^	.002^†^	.066	.821
No liver steatosis (*n* = 34)	4.1 (3.0─6.2)	6.1 (4.9─7.3)	3.9 (3.0─5.6)	2.9 (2.4─7.7)	.099	-	-	-
Liver cirrhosis (*n* = 49)	5.2 (3.3─7.6)	7.0 (5.8─10.8)	4.4 (3.2─7.4)	3.2 (2.7─6.9)	.012^†^	.006^†^	.022^†^	.569
No liver cirrhosis (*n* = 13)	5.7 (3.3─7.1)	10.3^‡^	4.3 (3.0─6.2)	8.2^‡^	na	-	-	-

Note.**—**Data are medians, with interquartile range in parentheses. *HCC*, hepatocellular carcinoma; *PDFF*, proton density fat fraction; *G1*, well-differentiated lesions; *G2*, moderately-differentiated lesions; *G3*, poorly-differentiated lesions; *na*, not applicable

^†^Denotes significant values

^‡^*n* = 1

### Steatotic lesions in cirrhotic livers

Liver cirrhosis was found in 47 of the 57 patients (74%), corresponding to a total number of 49 lesions. 11 of these lesions were classified as G1 (22%), 33 as G2 (67%), and 5 as G3 (10%). Similar to the overall sample, the fat fractions differed significantly between G1 (7.0%; IQR, 5.8─10.8%) and both G2 (4.4%; IQR, 3.2─7.4%; *p* = 0.006) and G3 lesions (3.2%; IQR, 2.7─6.9%; *p* = 0.022; Table 4 and Fig. 5c). Again, no difference was found comparing G2 and G3 tumors (*p* = 0.569). ROC analysis demonstrated that the fat fraction was also a discriminator between G1 and G2/3 lesions in this subsample, with an optimal threshold of 5.8% (sensitivity, 82%; 95% CI, 59─100%; specificity, 71%; 95% CI, 57─85%; Table 3 and Fig. 5d) and comparable performance to the overall sample (AUC of 0.79). Due to lack of sufficient numbers of lesions categorized as G1 (*n* = 1) and G3 (*n* = 1), no valid analysis could be performed for steatotic HCCs in non-cirrhotic patients.

### Lesion size and typical HCC imaging features by tumor grade

There were no significant differences between tumor grades regarding the maximum diameter of steatotic HCCs (*p* = 0.058). Lesions in the G1 category demonstrated the largest diameter (44.3 mm; IQR, 36.3─56.9 mm), followed by G3 lesions (41.2 mm; IQR, 30.0─84.9 mm) and G2 lesions (31.5 mm; IQR, 17.5─44.0 mm; see Table 2). Twenty-eight of the 62 lesions (45%) demonstrated the typical imaging features of HCC. Although the proportions of lesions with these features differed slightly between tumor grades (G1: 7 of 12 [58%]; G2: 19 of 44 [43%]; G3: 2 of 6 [33%]), these differences did not prove significant (*p* = 0.55).

### Correlation of PDFF and histological fat quantification

Of the 36 specimens obtained by resection, 28 were included in the histological assessment of intralesional fat content; five tissue sections were no longer available, and three were not evaluable due to severe damage to the sample. Correlation analysis revealed a statistically significant (*r* = 0.61; *p* < 0.01), but moderately positive correlation between PDFF and grading of intralesional fat content (see Table 5). The overall difference between the groups reached statistical significance (*p* = 0.019), whereas, in the pairwise comparisons, this was only observed for the differences between lesions classified as grade 2 or 3 (moderate or severe fatty degeneration), and those where very little or no fat was detected by light microscopy (*p* = 0.037 and *p* = 0.003). In 7 out of 28 samples (25%), no intralesional fat was recognized by the pathologist despite a positive finding in the PDFF map.

**Table 5:** Correlation of MR-derived proton density fat fraction (PDFF) and histological semiquantitative assessment of intralesional fat in 28 steatotic HCC specimens obtained by resection

Variable	Histological assessment of intralesional fat in steatotic HCCs				*p* value	*r* (95% CI)
	Grade 0 (very mild to none; < = 2%)	Grade 1 (mild; > 2─5%)	Grade 2 (moderate; > 5─10%)	Grade 3 (severe; > 10%)		
PDFF (%)	3.9 (2.8─4.3)	4.1 (3.1─7.7)	6.4^‡^ (4.1─8.4)	7.4^‡^ (4.4─10.3)	.019^†^	.61^†^ (.29─.80)
No of lesions* (*n* = 28)	9 (32)	7 (25)	5 (18)	7 (25)		

Note.**—** Except where indicated, data are medians, with interquartile range in parentheses. *p* values were obtained using Kruskal–Wallis test followed by Dunn´s multiple comparisons test. *Data are absolute frequencies, with percentages in parentheses. *PDFF*, proton density fat fraction; *HCC*, hepatocellular carcinoma; *r*, Spearman´s rank correlation coefficient; *CI*, confidence interval

^†^Denotes significant values

^‡^*p* < .05 versus grade 0

### R2* relaxometry for assessment of liver and lesion iron content

The median R2* in liver parenchyma was 35 s^−1^ (IQR, 31─43 s^−1^) at 1.5 Tesla and 52 s^−1^ (IQR, 48─72 s^−1^) at 3 Tesla, and in the HCC lesions, it was 27 s^−1^ (IQR, 21─31 s^−1^) at 1.5 Tesla and 35 s^−1^ (IQR, 27─41 s^−1^) at 3 Tesla. According to the recently published calibration curves [17], this translates to a median liver iron content of 0.7 mg/g dry tissue (IQR, 0.6─1.0 mg/g) and a median lesion iron content of 0.5 mg/g (IQR, 0.4─0.6 mg/g) with each individual reading below the upper limit of 1.8 mg/g for normal liver iron content. Therefore, no relevant bias of the PDFF measurements due to iron deposition is to be expected.

### Inter-reader agreement

The computed intraclass correlation coefficient was 0.96 (95% CI, 0.92─0.97; *p* < 0.01), indicating excellent agreement between the two readers on the PDFF measurements.

## Discussion

The present work suggests a relationship between the histologic tumor grade of steatotic hepatocellular carcinomas (HCCs) and intralesional fat content, as determined by MRI proton density fat fraction (PDFF) mapping. Well-differentiated steatotic HCCs (G1) showed significantly higher fat fractions (7.9%; interquartile range [IQR], 6.0─10.7%) than moderately (G2; 4.4%; IQR, 3.2─6.6%; *p* = 0.001)- and poorly (G3; 4.7%; IQR, 2.8─7.8%; *p* = 0.036)- differentiated steatotic HCCs, while the ROC analysis highlighted PDFF as a good discriminator (area under the receiver operating characteristic curve [AUC] of 0.81; *p* = 0.001) between G1 and less-differentiated steatotic HCCs (G2 and G3). Comparable results were observed for steatotic HCCs in cirrhotic livers. In steatotic livers, both the differences between G1 and G2/3 tumors and the diagnostic performance of PDFF to distinguish them were even higher than in the overall sample. Our findings suggest that quantification of intralesional fat in steatotic HCCs using MRI PDFF may be useful to predict tumor grade.

**Fig. 6:**
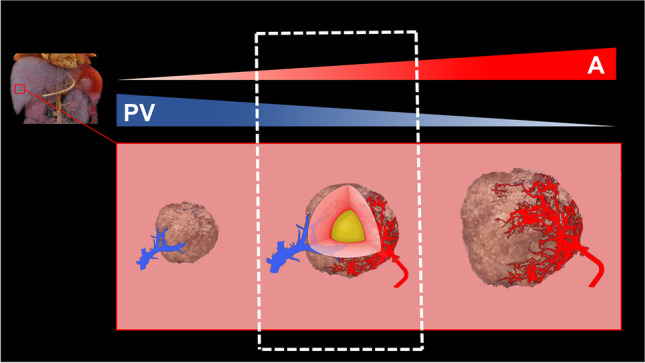
The concept of fatty metamorphosis during hepatocarcinogenesis due to transient hypoxia. In the course of dedifferentiation of HCCs or their precursors, the transition from portal venous dominant to arterial dominant blood supply leads to a narrow time window with relative hypoxia of the lesion-promoting steatogenesis (represented by the yellow core). PV = portal venous, A = arterial

The prevalence of steatotic HCCs is estimated to be between 10.0 and 37.2% [3, 11, 19–21]. It is commonly assumed that steatotic HCCs are equivalent to early tumor stages during hepatocarcinogenesis. Fatty change (a.k.a. fatty metamorphosis) of liver nodules occurs during hepatocarcinogenesis and is considered an indicator for the malignant transformation of premalignant lesions. The most widely accepted hypothesis for pathogenesis is the concept of transient hypoxia; during the transition between portal venous dominant supply and arterialization of the lesion, there is a time frame with incomplete neovascularization [3] resulting in focal hypoxia (Fig. 6). This promotes steatogenesis due to a decrease in the mitochondrial oxidation of fatty acids [22]. Our findings underline that fatty change occurs to a greater extent in earlier HCC stages, but not exclusively. Since it reflects the degree of intralesional hypoxia, an imbalance of lesion size and vascularization may also lead to fatty metamorphosis in advanced stages, as suggested by some authors [23, 24]. The higher fat content of steatotic HCCs in liver steatosis and the more pronounced differences between G1 and G2/3 lesions in this subgroup may indicate, that early-stage HCCs still exhibit an altered lipid metabolism similar to the rest of the liver, further contributing to fatty change.

Although the presence of intralesional fat in focal liver lesions is established in the LI-RADS classification as a characteristic favoring the diagnosis of HCC, its value as a diagnostic feature is of secondary importance and is based on older radiological-histopathological correlation studies. Only a few publications investigated non-invasive detection of intratumoral fat in HCCs by using chemical shift MRI [21, 25]; interestingly enough, intralesional fat has been proposed as a surrogate of tumor grade and good therapy response [5, 7, 26] with a lower rate of microvascular invasion [6]. Thus, accurate and convenient in vivo quantification of intralesional fat could facilitate an initial assessment of the degree of differentiation and prognosis in steatotic HCCs. Reliable and valid fat quantification with MRI PDFF mapping has already been established in diffuse liver disease and has almost eliminated the need for liver biopsy to diagnose and monitor liver steatosis [13, 27–29]. However, this technique has not been evaluated for focal liver lesions so far.

The moderately positive correlation of PDFF measurements and histopathological assessment of intralesional fat may be surprising at first glance, but in our opinion, it is inevitable due to the subjective and semi-quantitative nature of conventional histological analysis; as is known from studies on diffuse liver disease, histopathological fat quantification is compromised by very low interrater reliability, even among highly experienced pathologists [30]. This is to the point that other quantification methods, such as MR spectroscopy or PDFF mapping, are considered by many (including pathologists) to be more accurate [13]. The fact that the pathologist did not detect intralesional fat in 7 of 28 samples classified as steatotic by the PDFF measurement may reinforce this impression; if the histological diagnosis of steatotic HCC is synonymous with visualization of any macro- or microvesicular lipid droplets within the nodule, there remains the possibility of missing lipids that are undetectable due to the limited spatial resolution of light microscopy, but for which quantitative methods reflecting chemical composition (such as PDFF mapping) may be more sensitive. However, this should be investigated for focal liver lesions in future systematic studies with larger sample sizes.

We see our study as a necessary first step to establish tumor grade in steatotic HCCs (and PDFF as a tool for its non-invasive assessment) in precision medicine. While Zhou et al demonstrated the predictive value of histological tumor grade in HCCs without microvascular invasion [8], Sasaki et al came to the same conclusion for HCCs equal to or smaller than 2 cm [9]. By highlighting PDFF as a discriminator between well- and less-differentiated steatotic HCCs, our findings may stimulate further investigation into possible associations between intralesional fat content and treatment response in HCC patients, as this may have implications for optimizing future treatment stratification algorithms. It is particularly encouraging that discrimination with PDFF was feasible in patients with liver cirrhosis, as this subgroup is likely to benefit most from it for two reasons. First, the prospect of obtaining additional diagnostic and potentially prognostic information without the need for a biopsy is promising, as the risk of complications from invasive procedures is inherently higher. Second, these patients are often enrolled in HCC screening programs because of the higher probability of developing liver cancer; frequent MR imaging increases the chance of diagnosing early, well-differentiated HCC—a situation in which this technique may become relevant in the first place. Potentially, PDFF mapping could also play a role in the non-invasive distinction of certain histological HCC subtypes; although recent data by Mulé et al highlight the presence of fat in mass as determined by MRI as an independent predictor of steatohepatitic HCC [31], Cannella et al observed intralesional fat in the three most common subtypes (steatohepatitic, not otherwise specified and macrotrabecular-massive), making the detection of fat a rather unreliable imaging feature to discriminate these subtypes [32]. However, if there is a previously unknown association between intralesional fat content and HCC subtype, quantification with PDFF mapping could help overcome this problem and contribute to more accurate non-invasive subtyping, increasing its clinical utility in HCC diagnostics. A systematic study of the HCC subtypes could provide further insights.

Our study has several limitations. First, a selection bias must be considered. Since only lesions with histologic sampling were included, primarily earlier disease stages were examined, leading to an overrepresentation of G1 and G2 tumors. The small number of G3 lesions may have affected data quality and masked significant differences between G2 and G3 tumors. However, since this setting reflects clinical reality in which predominantly lesions in doubt and/or potentially curable stages are scheduled for biopsy or surgery, this rather underscores the validity and the importance of our findings. Second, there is a risk of sampling bias due to tumor heterogeneity. Nonetheless, more than half of the data were obtained by surgical resection, where sampling error is not a problem. Third, the threshold of 2.2% for PDFF as a definition of steatotic HCCs was empirical and extrapolated from the literature [18, 33]. To date, there is no clear definition for the histopathological diagnosis and grading of steatotic HCCs, nor are there studies that have systematically compared quantitative MRI with histopathological assessment of fat content in steatotic HCC specimens. However, as mentioned above, it is quite possible that PDFF mapping is superior to histopathology in terms of sensitivity and accuracy; further research on this topic is mandatory. The use of two different MRI field strengths (1.5 and 3 Tesla) also raises the question of the comparability of the PDFF outputs. Since this issue has already been addressed in phantom and patient studies without evidence of significant differences [14, 29, 34, 35] and we also observed similar effects in the groups for both field strengths, we consider the variance to be negligible. Eventually, despite a sample size of 62 lesions, this is still a small study. However, as this is the first systematic investigation of fat quantification with MRI in steatotic HCCs, we believe our results are not only interesting but may also influence future diagnostic and therapeutic algorithms in HCC.

## Conclusion

MRI proton density fat fraction mapping allows discrimination between well- and less-differentiated steatotic hepatocellular carcinomas. Thus, the investigation of intratumoral fat content as a potential prognostic indicator of treatment response is encouraged.

## Abbreviations

AUC: Area under the receiver operating characteristic curve
BCLC: Barcelona Clinic Liver Cancer
CI: Confidence interval
HCC: Hepatocellular carcinoma
IQR: Interquartile range
PDFF: Proton density fat fraction
ROC: Receiver operating characteristic
ROI: Region of interest

## Declarations

### Guarantor

The scientific guarantor of this publication is Patrick Arthur Kupczyk, MD.

### Statistics and biometry

Jennifer Nadal (Department of Medical Biometry, Informatics and Epidemiology, University of Bonn) kindly provided statistical advice for this manuscript. Furthermore, three of the authors have significant statistical expertise.

### Methodology

• retrospective

• diagnostic study

• performed at one institution
